# ACPSEM position paper: recommendations for a digital general X-ray quality assurance program

**DOI:** 10.1007/s13246-024-01431-y

**Published:** 2024-08-12

**Authors:** Timothy Ireland, Amanda Perdomo, Kam L. Lee, Adam Jones, Peter Barnes, Thomas Greig, Susan E. Reynolds

**Affiliations:** 1 Biomedical Technology Services, Queensland Health, Brisbane, QLD Australia; 2 Medical Imaging, Royal Children’s Hospital Melbourne, Parkville, VIC Australia; 3 Australian Radiation Protection and Nuclear Safety Agency, Yallambie, VIC Australia; 4 Medical Physics Department, Westmead Hospital, Westmead, NSW Australia; 5 Medical Physics, Austin Health, Heidelberg, VIC Australia; 6 Medical Imaging, John Hunter Hospital, Newcastle, NSW Australia; 7 Gamma Health Physics, Christchurch, New Zealand

**Keywords:** General X-ray, Quality assurance, Quality control, Digital radiography

## Abstract

This guideline has been prepared by the ACPSEM to provide a standardised quality assurance program to be used within General X-ray imaging environments. The guideline includes the responsibilities of various multidisciplinary team members within medical imaging facilities. It must be noted that the listed tests and testing frequencies are not intended to replace or become regulatory requirements. Implementing a quality assurance program as outlined in this position paper is there to ensure best practice for imaging facilities by providing a framework to establish and monitor correct equipment performance. The current document has been produced through an extensive review of current international practices and local experience within the Australasian healthcare environment. Due to the constant evolution of digital radiographic equipment, there is no current consensus in international quality assurance guidelines as they continue to be adapted and updated. This document describes the current state of the use of digital General X-ray equipment in the Australasian environment and provides recommendations of test procedures that may be best suited for the current medical imaging climate in Australasia. Due to the everchanging developments in the medical imaging environment and the ability of new technologies to perform more complex tasks it is believed that in the future this document will be further reviewed in the hopes of producing a more globally agreed upon standard quality assurance program. Any such adjustments that are deemed to be necessary to Version 1.0 of this document will be provided in electronic format on the ACPSEM website with a notification to all parties involved in the use of digital General X-ray equipment. This guideline does not provide detailed methodologies for all the quality control tests recommended as it is it is expected that the professionals implementing aspects of this quality assurance program have the working knowledge and access to appropriate resources to develop testing methodologies appropriate for their local imaging environment.

## Introduction

### Background

There is currently no standardised comprehensive quality assurance (QA) program for general radiographic equipment in Australia and New Zealand, however, use of general radiographic equipment is ubiquitous throughout the region.

A standardised QA program allows for greater understanding and comparison of equipment performance, identification of outlier equipment performance, and identification of performance trends. Overall, a standardised QA program will improve the quality of clinical services within Australia and New Zealand.

It is the intent of the Australasian College of Physical Scientists and Engineers in Medicine (ACPSEM) to establish a set of evidence-based recommendations for a best-practice QA program for digital general radiographic practices. A general X-ray Working Party was established by the ACPSEM to collate and review current QA practices, both nationally and internationally. This review has informed the recommendations of this guideline for a general X-ray QA program.

### Scope of this document

The scope of this guideline is to recommend an evidence-based QA program for digital general X-ray systems within Australia and New Zealand. The QA program should be maintained in consultation with a Qualified Medical Physics Specialist (QMPS) in radiology physics, who will work within a multidisciplinary clinical team that may consist of physicists, radiographers, engineers and radiologists. This guideline is not designed to be adopted as a mandatory requirement for all regulatory bodies throughout Australia but is rather a guide for best practice in terms of QA in general radiography and is there to guide individual facilities and multidisciplinary teams as to what QA is recommended to be performed. The QA program for a digital general X-ray system contains many elements including procurement, acceptance testing, commissioning, routine quality control (QC) and facility QC. The focus of this paper is primarily to provide guidance on the elements of the QA program that are related to ensuring that optimal equipment performance of the X-ray equipment is maintained.

This guideline is intended to provide:

- A summary of the roles and responsibilities of the medical physicist in general X-ray QA.
- Recommendations for acceptance testing, and commissioning of digital general X-ray imaging equipment performed by a medical physicist.
- Recommendations for routine QC of digital general X-ray imaging systems performed by a medical physicist.
- Recommendations for routine performance evaluation of digital general X-ray imaging systems performed by facility staff.

### Terminology

This guideline is non-regulatory in nature and consists of recommendations to conform to contemporary best practice. Within this framework, there are some tests that must be performed at the specified frequency to ensure compliance with best practice. These tests are defined as “recommended” components of the QA program. Within this framework there are some tests that may be performed as troubleshooting measures or would only be recommended under certain circumstances. These tests are defined as “optional” components of the QA program and should be performed as required at the discretion of a QMPS. The terms “medical physicist” and “QMPS” are used throughout this document to refer to a medical physicist listed on the professional register of Qualified Medical Physics Specialists.

For the purposes of this guideline, different components of the QA program have the following definitions:

**Table:** 

Test type	Frequency	Performed by	Purpose
Acceptance testing	At purchase	Medical Physicist	Verification that a piece of equipment (including software and peripheral equipment) has been supplied as specified during procurement. Acceptance testing includes verification that system meets minimum safety and regulatory standards
Commissioning	Prior to clinical use	Medical Physicist	Verification that equipment has been installed, configured, and calibrated in a manner that is fit for clinical use. Performance baselines for future routine testing are also established during the commissioning process. Commissioning is typically performed after installation, manufacturer’s internal tests, and acceptance testing, but before clinical use
Routine quality control	Periodically (infrequently e.g. annually)	Medical Physicist	Tests to ensure that the performance of a system remains within acceptable criteria. Testing frequency depends on the potential clinical implication and the likelihood of the system performance changing
Performance checks	Periodically (frequently e.g. monthly)	Facility Staff	A small subset of potentially simplified Routine Quality Control tests which are undertaken more frequently than routine QC. These checks are intended to be simple and quick to undertake but act as indicators for change in important system performance or function

Replacement components e.g. X-ray tubes and image receptors must also be subject to relevant acceptance and commissioning testing.

## Roles and responsibilities of the medical physicist

The Qualified Medical Physics Specialist is responsible for the oversight of the multidisciplinary clinical team maintaining the QA program for general X-ray systems. The primary role of the QMPS within this multidisciplinary team is to ensure the safe use of radiation whilst maintaining optimal equipment performance. It is the position of the ACPSEM, that to fulfil this role the QMPS must have the following responsibilities within the QA program:

- Provision of expert advice in relation to equipment procurement, radiation safety and any developments in technology and/or clinical techniques.
- Acceptance testing and commissioning of new equipment to confirm that the system is fit for clinical purpose and to establish appropriate baselines.
- Confirm that all local regulatory safety requirements are satisfied.
- Routine QC performance testing.
- Oversight, facilitation and review of facility QC procedures
- Provide recommendations that may improve the optimisation of radiation dose and clinical image quality.
- Maintain records of data collection for analysis and review.

## Facility quality control procedures

### Introduction

Facility QC procedures are essential for ensuring high-quality diagnostic images. The facility QC procedures are to be performed by a facility representative (e.g. a radiographer or a medical physicist) and supervised by a QMPS [1, 2].

Appendix 1 lists the routine QC procedures with key procedure elements and relevant tolerances.

### Procedure recommendations

#### Detector calibration

*Frequency: As specified by manufacturer*

*Rationale*

Modern digital imaging systems have the capability to correct for many factors that can lead to non-uniform images. Two of the major sources of image non-uniformity are detector element inhomogeneous response and X-ray output.

Both factors are addressed by calibrating the image receptor gain response to create a calibration map that can be applied to every image to ensure that image variation is due to clinical anatomy, and not due to X-ray system components.

Over time, this calibration map can become less effective and may need to be re-calibrated. The test methodology and frequency of calibration is best specified by each X-ray image receptor manufacturer.

*Performance criteria*:

As specified by manufacturer.

#### System constancy

*Frequency: Monthly*

*Rationale*

There are several indicator metrics that can be used to identify underlying equipment performance degradation. Frequent monitoring of these metrics with appropriate tests can identify any equipment performance degradation before clinical images are impacted [3]. Consistent and regular performance of these tests, with appropriate documentation outlining the testing methodology can improve the sensitivity of these tests.

The system constancy test identifies consistent performance of key imaging chain components such as the X-ray tube, KAP meter, AEC and digital image receptor. This test can be performed by taking a single AEC driven exposure for each image receptor, with reproducible set-up and technique factors.

*Performance criteria*

For each image the following metrics should be recorded and compared to baseline values [3] (Table 1):

**Table 1:** Constancy test variables and tolerances

Metric	Tolerance
X-ray tube current–time product (mAs)	≤ ± 20% of baseline; or ≤ ± 0.2 mAs whichever is greater
Kerma-Area-Product* (KAP)	≤ ± 15% of baseline
Exposure Index* (EI)	≤ ± 20% of baseline***
Mean Pixel Value**(MPV)	≤ ± 20% of baseline***
Standard Deviation (SD) of MPV*	≤ ± 20% of baseline***

*If metric is available, or alternative appropriate metric. Kerma-Area-Product and Dose-Area-Product are largely used synonymously and is dependent on manufacturer preference for naming convention

**Mean Pixel Value measurement should be taken in the central portion of the image to avoid pixel value variation with heel-effect. Consistent placement of Region of Interest will elicit more consistent results. Analysis must be performed on “For Processing” images

***For systems where linear pixel values and EI are not available, tolerances will not be applicable and appropriate tolerances should be developed by a QMPS and substituted

#### Image uniformity and artefact evaluation

*Frequency: Monthly*

*Rationale*

Modern digital imaging systems have the capability to correct for many factors that can lead to non-uniform images. As such, a flat-field image should be very uniform in appearance.

A visual inspection of the images from “System constancy” section must be performed with a narrow window width and appropriate level setting to assess the images for general uniformity and possible image artefacts of clinical significance. Windowing, zoom and pan functions should be utilised.

*Performance criteria*

The image must be uniform and free from significant artefacts such as those listed in the image uniformity and artefact evaluation criteria in Appendix 1.

#### Modality display QC

*Frequency: Quarterly*

*Rationale*

Secondary displays are those used for viewing medical images for purposes other than for providing a medical interpretation. Modality displays, also known as secondary displays, are utilised during acquisition and quality checks of medical images. The performance of these displays directly impacts image appearance used for interpretation, so it is imperative that they meet minimum performance standards.

The TG18-QC test pattern has been widely adopted as the default qualitative assessment pattern, replacing the older SMPTE pattern. It is recommended that the TG18-QC pattern be used rather than the SMPTE pattern which is primarily CRT-centric. In some more modern systems, a TG270-sQC (simple QC) may be the pattern of choice leading into the future [4].

*Performance criteria*

All criteria identified in the modality display QC checklist in Appendix 1 are met for the TG18-QC test pattern.

#### Mechanical and peripherals inspection

*Frequency: Quarterly*

*Rationale*

Radiographic equipment undergoes the same wear and tear as any mechanical device, As such, it is important to periodically inspect radiographic systems to ensure that there are no hazardous, inoperative, misaligned or improperly operating components.

An overall mechanical and peripherals inspection of the general X-ray system and associated components must be performed. Particular attention should be given to components that are used frequently. The inspection should include all relevant components indicated in Appendix 1 at the indicated frequency.

*Performance criteria*

All relevant components of the mechanical and peripherals inspection checklist in Appendix 1 have been inspected and confirmed to be operating correctly.

#### X-ray/light field alignment

*Frequency: Quarterly*

*Rationale*

The purpose of this test is to ensure that the X-ray field, light field and image receptor are aligned. Misalignment of these components can lead to collimator cut-off, leading to missed tissue or the patient being unnecessarily exposed beyond the image receptor.

This test is particularly important for mobile X-ray systems where physical damage can cause misalignment.

*Performance criteria*

The maximum variation in X-ray field to light field should be ≤  ± 1% of the focus-to-Image detector distance (FID) [5].

#### Rejected image analysis

*Frequency: Quarterly*

*Rationale*

Repeated and rejected images are a source of unnecessary radiation exposure and create inefficiency within a medical imaging service. Repeated and rejected images cannot be eliminated but can be reduced to a minimum with an effective analysis system.

While digital radiography systems have made image acquisition easier compared to film-screen systems, the option to repeat or reject an image is also easier. Rejected images may risk being unaccounted for if an active monitoring program is not in place [6, 7]. A reject-retake analysis system must be established. If available, automated methods should be used to collect and analyse data [8].

Rejected image data should be extracted monthly and prior to a service to ensure it is not inadvertently removed. Data analysis should occur quarterly but must occur at least annually.

The rejected image rate must be calculated as:

1 $$ Rejected Image Rate = \frac{Number of rejected images}{{Total number of images acquired}} $$

The rejected image rate threshold is dependent on the type of service. For example, services with student radiographers or a complex examination workload, can accept a higher reject rate. A reject rate of 8 ± 2% is considered appropriate for a typical service using digital radiography [7, 9–13]. Special consideration should be given to paediatric radiography. In this case, a reject rate of 5 ± 2% is more appropriate [6, 11]. Not only will reject rates vary depending on the service provided, but also on the radiographic procedure. For example, it is expected that a PA Chest procedure will have a lower reject rate compared to a more complex procedure [11, 13]. For this reason, rejected images should be stratified into specific categories with appropriate investigation thresholds.

An effective reject image analysis program will not eliminate rejected images but will optimise the system. If the reject analysis system is not optimised there remains the possibility that inadequately acquired images may be sent for clinical interpretation. If that were the case, radiologists require a mechanism to identify and reject such images as part of the analysis.

The results from rejected image analysis should be reviewed, documented, and kept for reporting.

*Performance criteria*

Reject rate upper limit should be 10% for adults and 7% for paediatrics.

Reject rate lower limit should be 5% for adults and 3% paediatrics.

#### General X-ray quality meeting

*Frequency: Quarterly*

*Rationale*

Regular general X-ray review meetings must occur with documented minutes. These meetings should occur on a quarterly basis.

The following facility staff should attend these meetings (where practical):

- Administrator (e.g. chief radiographer or appropriate delegate)
- Senior radiographer
- Clinical representative (e.g. Radiologist, Radiology registrar)
- Technical representative (e.g. service engineer, maintenance contract manager)
- Medical Physics representative (e.g. QMPS)
- Radiation safety representative (e.g. Radiation Safety Officer)

The following agenda should be included at these meetings:

- Review of facility QC
- Review of any relevant incidents
- Review of any image quality complaints from clinical images
- Review of repeat/reject analysis
- Review of radiation dose audits/trends
- Review of protocol management

Additionally, the meeting should aim to identify or correct atypical performance and areas for QA improvement.

#### Data validation

*Frequency: Annuallyand with software reload/upgrade*

*Rationale*

With increased integration and use of data in healthcare, it is essential that critical DICOM metadata displayed on the image is accurate and consistent. A periodic check of the accuracy of displayed DICOM data must be performed, particularly following changes to major computer components or software.

It is recommended that data validation is performed remotely from the acquisition workstation (e.g. PACS or third party DICOM viewer) to ensure that data is transferred correctly.

*Performance criteria*

All data elements identified in the Data Validation checklist in Appendix 1 must be populated with correct information in the DICOM header.

Patient demographic and facility data should be checked for accuracy.

#### Dose and EI audit

*Frequency: Annually*

*Rationale*

The Exposure Index (EI) for digital radiography is defined by the IEC [14]. It is an indication of the image receptor incident air kerma, which in turn is an indication of the signal-to-noise ratio (SNR) of the final image.

Each examination protocol should have a target EI, which is determined by the target image receptor dose. By comparing the displayed EI to the target EI, most modern digital X-ray systems will generate a Deviation Index (DI). As the DI increases, the displayed EI deviates further from the target EI. DI is an alternate method to monitor consistent and appropriate image receptor air kerma.

Radiation exposure information can be recorded and extracted in many ways from digital X-ray systems. If available, the best monitoring metric is the Kerma Area Product (KAP, P_KA_). It is located within the DICOM header element (0018,115E) “Image and Fluoroscopy Area Dose Product”, the Radiation Dose Structured Report (RDSR) or within manufacturers’ exposure logs.

There are many methods to acquire radiation dose data, such as manually sampling records for all or specific examinations, sending images to a parallel DICOM node for data parsing and storage, or utilising large scale commercial automated data collection and analysis systems. Each of these methods has advantages and disadvantages. The method most appropriate for the facility should be implemented.

Where possible, the following data should* be extracted from all generated images [8]:

1. Machine ID
2. Operator ID
3. Examination type
4. Body part
5. View
6. Exposure Index
7. Radiation dose metric (KAP)**
8. Presentation intent (i.e. processed image)
9. Radiation field size
10. Tube voltage (kV_p_)
11. Tube current–time product (mAs)
12. Reference dose (i.e. target air kerma for a specified EI)

*Not all systems will be able to generate all metrics. Where this is the case, collect as much as possible.

**Note that specific attention must be given to the units of the radiation dose metric.

This data can be further subdivided, according to the machine, examination, view and operator, to identify the distribution of radiation dose and EI within a facility. This type of audit should be conducted at least annually, with patient or image receptor dose trends monitored over time, and where possible, between systems and other facilities. The audit should include any investigation and corrective action taken. All results must be reviewed, documented, and recorded.

#### Maintenance and fault logging

*Frequency: As required/Ongoing*

*Rationale*

Any maintenance work or equipment fault must be noted in a logbook so that changes to the equipment can be monitored over time. A separate logbook should be kept for each imaging system.

It is important to ‘close the loop’ on fault reports. This means that in addition to recording the fault, a note must be made to record any action taken by an engineer and any QA carried out to confirm that the problem has been resolved. It is useful to keep the engineer’s service reports, either alongside the fault log or in a separate folder.

It is recommended that, where possible, logbooks are kept electronically to allow for ease of access and data retention.

## Medical physics: acceptance, commissioning and routine quality control

### Introduction

Acceptance testing is essential to ensure that equipment meets minimum safety standards and has been supplied as specified during procurement. Commissioning ensures that the equipment has been installed, configured, and calibrated in a way that will be optimal for clinical use, and routine QC ensures that the system performance remains within tolerable limits from commissioning for the lifetime of the equipment. These tasks are not onerous and ensure that a facility gets value-for-money and optimised diagnostic sensitivity when using complex radiographic equipment.

Although there are many tasks that are nominally performed by a medical physicist, it is expected that this should be interpreted as “under the guidance of a qualified medical physics specialist”. For example, many tests required for acceptance testing would be conducted by manufacturer representatives during installation. It is not expected that a QMPS would re-perform these tests if they are confident that the performance of the system has been appropriately characterised. However, it is expected that the QMPS will review the results and retain a record.

During commissioning, the medical physicist must facilitate the implementation of the facility quality control program. This may include assisting with enabling equipment features such as access to exposure logs, establishing testing protocols and setting baseline performance values.

For all below tests, it is recommended that a thorough investigation of system performance is undertaken during acceptance testing, with “spot checks” of system performance conducted for the more commonly used clinical factors during routine QC.

Appendices 2, 3 and 4 list the Acceptance, Commissioning and Routine QC testing recommendations respectively.

### X-ray tube and generator tests

The accuracy and consistency of X-ray tube output is fundamental to the production of high-quality diagnostic images. While it is expected that modern X-ray tubes and generators can easily meet and exceed these standards, it is essential that these components are routinely evaluated to ensure continued high-quality performance.

#### Tube output repeatability

*Frequency: Acceptance and 2-yearly thereafter*

*Rationale*

X-ray tube output is a fundamental basis on which system performance is based. Inconsistent X-ray tube output will result in unpredictable patient exposure and image quality.

*Performance criteria*

The coefficient of variation of the X-ray output from a series of not less than 5 consecutive exposures must not exceed 0.05.

#### Tube output linearity

*Frequency: Acceptance and 2-yearly thereafter*

*Rationale*

Tube output should linearly increase with mA and mAs. This allows the X-ray operator to estimate the radiographic technique factors for clinical exposures. Non-linear X-ray tube output could result in under or over-exposed images, as well as unintended patient exposures.

*Performance criteria*

For any two mA (with a fixed exposure time) or mAs settings:

$$ \left| {{\text{X1 }}{-}{\text{ X2}}} \right| \, \le \, 0.{1 }\left( {{\text{X1 }} + {\text{ X2}}} \right) $$

X1 = X-ray output per mAs (or mA for fixed exposure time) at setting 1.

X2 = X-ray output per mAs (or mA for fixed exposure time) at setting 2.

The absolute value of $\left| {{\text{X1 }}{-}{\text{ X2}}} \right|$ divided by (X1 + X2) must be ≤ 0.1 and this is referred to as the linearity coefficient (LC).

#### Tube output

*Frequency: Acceptance and 2-yearly thereafter*

*Rationale*

X-ray tube output should be within a typical range to ensure that exposure duration is not excessively long or short and is predictable by an operator.

Excessively high or low X-ray tube output is likely the result of related X-ray tube issues such as insufficient filtration, X-ray tube deposition or a damaged anode.

*Performance criteria*

X-ray tube output is expected to be in the range of 20–80 µGy/mAs at 1 m from the focal spot using 80 kV_p_ and ≥ 2.5 mm Al total filtration.

#### Tube voltage accuracy

*Frequency: Acceptance and 2-yearly thereafter*

*Rationale*

X-ray tube voltage (kV_p_) is a primary determinant of patient exposure and image contrast. The delivered kV_p_ must match the selected kV_p_ to ensure that the resultant exposure is as intended by the X-ray operator.

*Performance criteria*

The kV_p_ accuracy for kV_p_ settings across the clinical range must not exceed ± 5%.

#### Filtration

*Frequency: Acceptance and 2-yearly thereafter*

*Rationale*

X-ray beam filtration plays a significant role in the shaping of the X-ray beam spectrum. A beam that has insufficient filtration can result in excessive radiation exposure to the patient, and a beam that can reduce image contrast.

*Performance criteria*

The Half-Value Layer (HVL) measured at 80 kV_p_ must be ≥ 2.9 mm Al.

Half-Value Layer measured across the range of clinically used kVps should meet IEC 60601-1-3 requirements [15].

For X-ray systems installed prior to 2008, HVL must be > 2.3 mm Al at 80 kV_p_.

#### Timer accuracy

*Frequency:*

*Acceptance only for crystal-controlled timers (i.e. timer mechanism in modern systems)*

*Acceptance and 2-yearly thereafter for resistor–capacitor (RC) timers*

*Rationale*

X-ray timers control the length of the radiation exposure to the patient and image receptor. As such, any error in the timer will have a linear impact on both the length of time that the patient is exposed for, as well as the patient dose and image receptor air kerma.

*Performance criteria*

Measured time must be within ≤ 10% of indicated time for times ≥ 100 ms. Measured time must be within ± (10% + 1 ms) for times < 100 ms. Test should not be performed for times any lower than 20 ms as the measurement error of the equipment is too great.

#### Leakage radiation

*Frequency: Acceptance and 2-yearly thereafter*

*Rationale*

Leakage radiation transmitted through the protective housing of the X-ray tube and collimator (source assembly), and including scattered radiation produced within the source assembly, can result in unnecessarily high patient or operator dose.

At acceptance, tube change and system relocation, a thorough test using image receptors, if possible, placed around the source assembly to detect possible areas of higher-than-expected radiation is recommended. If image receptors or gafchromic film are not available then a suitable survey meter can be used as per for routine tests as a surrogate. For routine tests, spot checks (usually 6 measurements around the housing) using a suitable survey meter is recommended.

*Performance criteria*

Leakage radiation must be ≤ 1 mGy per hour at a distance of 1 m from the focus at the maximum nominal voltage and maximum continuous current specified by the manufacturer for that tube in that housing.

#### Light/X-ray field alignment

*Frequency: Acceptance and 2-yearly thereafter*

*Rationale*

Correct light and X-ray field alignment will ensure that the area intended to be irradiated is fully irradiated, and no additional tissue is irradiated. This means no missing tissue and no unintentional tissue irradiation.

*Performance criteria*

For each focus and each X-ray field boundary, the edge of the X-ray field must be ≤ 1% of the FID within the light field.

For each focus and each X-ray field boundary, the edge of the X-ray field must extend ≤ 1% of the FID beyond the light field.

#### Light/X-ray field congruence

*Frequency: Acceptance and 2-yearly thereafter*

*Rationale*

Alignment of the centre of the X-ray and light fields will ensure that focal spot positioning over the area of interest is maintained, and off-focus blurring is minimised.

*Performance criteria*

Either:

Centre of X-ray and light fields should coincide to ≤ 1% of FID.

Centre of X-ray and light fields must coincide to ≤ 1.5% of FID.

#### OR

Centre of X-ray and light fields must coincide to within ± 3.8°. This corresponds to 10 mm for a 20 cm test object with opaque beads at the top and bottom of the phantom with an FID of 100 cm.

#### X-ray to image receptor alignment

*Frequency: Acceptance and 2-yearly thereafter*

*Rationale*

Modern radiographic systems have automated and/or manual mechanisms in place to align the X-ray tube to fixed image receptors. Misalignment of these components will lead to anatomical cut-off and unnecessary patient exposure.

*Performance criteria*

When in a detent location, with the maximum selectable field of view (FOV), the X-ray field must extend to the edge of the active detector region and must extend beyond the image receptor ≤ 1% of the FID.

#### Light field illuminance

*Frequency: Acceptance and 2-yearly thereafter*

*Rationale*

The light field must be of sufficient luminance to enable accurate definition of the X-ray field during patient set-up.

*Performance criteria*

Illuminance should be > 160 lx at 1 m from the focal spot.

Illuminance must be > 100 lx at 1 m from the focal spot.

### Automatic exposure control (AEC)

The Automatic Exposure Control calibration is one of the most significant contributors to patient radiation exposure and diagnostic quality of resulting images. As such it is important to ensure that the AEC sensitivity is set to obtain appropriate exposure level to the image receptor across the range of clinically used beam qualities.

Prior to commissioning the AEC system, it is important to review manufacturers guidance on their specific AEC system. This review should take place with seniorradiographers from the department to determine important factors such as:

- Patient exposure/image receptor incident air kerma
- Typical image noise levels
- AEC sensitivity as a function of tube voltage

In the absence of any guidance from manufacturer or local departments, it is recommended that AEC sensitivity is measured in terms of Exposure Index. This is chosen as it is a practical metric available on all modern X-ray equipment and is correlated strongly with image receptor incident air kerma and resultant image noise. Other metrics that could be considered are image receptor incident air kerma, mean pixel value, signal-to-noise ratio or signal-difference-to-noise-ratio.

Prior to using any of these metrics, appropriate testing should be conducted to ensure their accuracy (e.g. Signal Transfer Properties (STP) and EI relationship and accuracy to image receptor incident air kerma).

There is no single correct Exposure Index to universally apply across General X-ray systems due to the unique equipment setup in terms of image receptor material, beam quality and clinical application. As such, it is recommended that the image receptor incident air kerma defined by the image receptor manufacturer, is considered as a starting point for discussion with the local department.

#### AEC sensitivity

*Frequency: Commissioning and 2-yearly thereafter*

*Rationale*

The sensitivity of the AEC chambers is set to deliver the planned image receptor incident air kerma. An outcome of this is that the AEC sensitivity is the primary determining factor of image noise and patient exposure.

*Performance criteria*

Under clinical exposure conditions, the measured image receptor incident air kerma should be within ± 20% of the target air kerma, and be ≤ 3 µGy. Investigations must be undertaken if the image receptor incident air kerma is > 5 µGy.

#### AEC repeatability

*Frequency: Acceptance and 2-yearly thereafter*

*Rationale*

AEC systems are expected to consistently deliver a pre-defined image receptor exposure. With a consistent amount of attenuation there should be minimal variance in the way that an AEC system responds.

*Performance criteria*

For five consecutive exposures, the coefficient of variation of the image receptor incident air kerma must not exceed 0.05 [16].

#### AEC reproducibility

*Frequency: Commissioning and 2-yearly thereafter*

*Rationale*

Once commissioned, the AEC sensitivity should remain constant over time to ensure that image receptor incident air kerma and resultant image quality does not drift.

*Performance criteria*

Using a consistent attenuator between exposures, post-exposure mAs under AEC control should be within ± 20% of baseline value for the most commonly used clinical protocol.

#### kV_p_ variation

*Frequency: Commissioning and 2-yearly thereafter*

*Rationale*

The image receptor incident air kerma should follow the relationship defined in collaboration with the equipment manufacturer and clinical department (see “Automatic exposure control (AEC)” section Introduction). Otherwise, the image receptor incident air kerma should remain constant across the range of clinically used tube voltage settings to ensure a constant image noise.

*Performance criteria*

Image receptor incident air kerma must not vary by more than 20% across a range of clinically used tube voltages.

Exposure index can be used to determine image receptor input air kerma for this test.

#### AEC termination

*Frequency: Acceptance and 2-yearly thereafter*

*Rationale*

Where the AEC system is used to control and terminate X-ray exposures, there must be failsafe systems in place to ensure that excessive exposures are limited in the event of AEC malfunction or incorrect setup (e.g. AEC not being irradiated).

*Performance criteria*

Under AEC control, exposure greater than 600 mAs must not be allowed [16].

#### AEC guard timer

*Frequency: Acceptance and 2-yearly thereafter*

*Rationale*

An AEC guard timer is used to manually limit the mAs that an X-ray exposure can reach. When available, the set guard timer must accurately terminate the exposure at the set mAs.

*Performance criteria*

If a guard timer is available, AEC controlled exposures must terminate before or at the set AEC guard timer.

#### AEC lateral chambers

*Frequency: Acceptance and 2-yearly thereafter*

*Rationale*

Lateral chambers are available with AEC systems to allow users to select an image receptor incident air kerma for specific regions of anatomy that may be offset from the central AEC chamber (e.g. lung field in a chest X-ray).

*Performance criteria*

The AEC system must control exposures such that the displayed mAs does not vary by more than 10% between individual AEC chambers for a consistent attenuation and set tube voltage.

#### AEC indication

*Frequency: Acceptance*

*Rationale*

X-ray systems can have several AEC chambers. The desired AEC chamber is carefully selected to ensure appropriate patient exposure and image quality based on patient positioning and active image receptor. Incorrect AEC chamber selection can lead to incorrect patient exposure and/or inadequate image quality. It is essential for a user to be able to identify the active AEC chamber prior to making an exposure.

*Performance criteria*

There must be visible indication of:

- The image receptor selected; and
- The AEC chamber(s) that are active

The indicated selections must match the active chambers.

### Image receptor

The image receptor in digital radiography takes the incident X-ray exposure and converts this to a digital image for diagnostic review. Problems in the conversion process can be subtle and undermine the diagnostic sensitivity of an image receptor by altering things like the noise patterns, sharpness, or uniformity in the diagnostic image.

Digital image receptor testing, while obviously important, has not been in widespread practice for as long as X-ray tube and generator tests. As such many tests are not required by local regulation and have been listed as optional in this guidance document. Optional tests in this space should not be considered unimportant, rather, they have been nominated as such to allow for flexibility in their implementation due to their relative novelty in many Australian and New Zealand practices.

All images used in this section refer to raw images, which is the same as original data as defined in IEC 62220-1-1 [17], and if possible “linearised” images. It is advisable to have a detector calibration performed prior to performing the tests in this section if one has not been performed recently.

#### Signal transfer properties (STP)

*Frequency: Acceptance and 2-yearly thereafter*

*Rationale*

Before any measurements can be made using image receptor pixel values, the relationship between pixel value and image receptor incident air kerma (STP) must be established. This allows for pixel value variations to be compared back to variations in sensitivity to air kerma.

If the STP is not linear in response to image receptor air kerma, then an inverse function of the STP must be applied to any images prior to quantitative analysis.

To measure the STP, it is recommended that RQA5 beam quality [18] is used to make 5 exposures across the dynamic range of the image receptor (e.g. 1/3—3 × the typical image receptor incident air kerma).

Some image receptors are integrated into a Bucky/housing that may incorporate a fixed grid. The air kerma measurements will overestimate the image receptor incident air kerma values in these systems unless an appropriate correction is made.

*Performance criteria*

Relationship between image receptor incident air kerma and mean pixel value (MPV) must be verified as simple (e.g. linear, log or power) with R^2^ > 0.99.

#### Exposure index

*Frequency: Acceptance and 2-yearly thereafter*

*Rationale*

The exposure index (EI) is an identification of the image receptor incident air kerma within a region of interest of an image.

The accuracy of the EI is important as this is a quantifiable metric that can be used to ensure continued appropriate function of the AEC of a General X-ray system. Additionally, clinical sites can set target EIs for all exposures and the deviation index (DI) will be calculated based on the difference between the displayed EI and the target EI. Large differences in these values can cause users to repeat clinical exposures.

While IEC 62494-1 [14] defines the EI in significant detail, at the date of publication there were still several prominent X-ray equipment manufacturers that do not strictly adhere to this formalism.

Prior to performing this test, it is recommended that manufacturer formalisms are understood. Particularly:

1. The expected relationship between image receptor incident air kerma and EI
2. The beam quality under which this is established (e.g. RQA5 [18])
3. The manufacturers method of determining the region of interest from where the EI is calculated

*Performance criteria*

Using RQA5 [18] beam quality, EI must be within 20% of the IEC defined EI relationship:

EI = 100 × image receptor incident air kerma (µGy).

#### Artefact evaluation

*Frequency: Acceptance and 2-yearly thereafter*

*Rationale*

Image artefacts and non-uniformities can degrade the diagnostic sensitivity of clinical images. It is therefore important to identify any artefacts and image non-uniformities. Image artefacts can originate from any component of the imaging chain (X-ray tube, collimator, patient support, grid, image receptor, post-processing, detector uniformity calibrations).

*Performance criteria*

An exposure of a uniform attenuator with a typical clinical image receptor incident air kerma must be free from clinically significant artefacts.

#### Uniformity

*Frequency: Acceptance and 2-yearly thereafter*

*Rationale*

Non-uniformities in digital images can mimic or obscure clinical pathology (e.g. shading, splotches/shadows). All digital image receptor manufacturers have therefore developed calibration methods to remove non-uniformities from their clinical images.

As this is a quantitative test, it is recommended that the STP is used to determine the image receptor incident air kerma by applying an inverse STP equation to the measured MPVs.

It is expected that the greatest variance in pixel values will be between the centre of the FOV and the peripheries of the image. As such, regions of interest should be placed centrally and in each of the four image corners in a full-field flat field image using a uniform attenuator.

*Performance criteria*

The maximum deviation between the STP-corrected central MPV and any other MPV should be less than 10%.

#### Variance image

*Frequency: Commissioning and 2-yearly thereafter*

*Rationale*

A variance image looks for pixel value variation within a relatively small region of interest that is iterated across the whole image. The variance image displays the variation of the pixel values rather than the pixel values themselves. The variance image is very sensitive to spatial artefacts and non-uniformities (e.g. physical detector damage, dead detector elements, detector hydration etc.).

*Performance criteria*

No significant variance defects are visible. Image comparable to baseline.

#### Defective detector elements

*Frequency: Acceptance and 2-yearly thereafter*

*Rationale*

Digital image receptors are composed of millions of detector elements (DELs) arranged into lines/rows and banks. Detector elements can malfunction in any of these configurations (individual DELs, rows, banks) resulting in signal loss in that area. Modern image receptors are able to mask many defective DELs in a way that does not significantly compromise diagnostic sensitivity, however there is a limit to how much data can be interpolated.

Many manufacturers will be able to provide a “defect map” with the location and quantity of defective detector elements. Most or all manufacturers will also have a tolerance for the number and configuration of dead detector elements that they consider acceptable.

*Performance criteria*

No clinically significant clusters of defective detector elements.

Number of defective detector elements within manufacturers specification.

#### Stitching

*Frequency: Commissioning and 2-yearly thereafter*

*Rationale*

Large format image receptors are comprised of smaller panels. To create a single clinical image, the signal from each of the smaller panels is stitched together. Where these panels are stitched together it is possible for there to be a slight mismatch or gap which can be visible in clinical images.

Stitching is most easily assessed by imaging a fine grid or mesh object and following the straight lines along their length to identify any discontinuity or mismatch as they transition between detector panels.

*Performance criteria*

No clinically significant stitching visible in images.

#### Image retention

*Frequency: Commissioning and as required thereafter*

*Rationale*

After an X-ray exposure there may be incomplete erasure of detector element signal, particularly in regions of the image receptor with unattenuated exposure. This may lead to previous exposure information being superimposed onto subsequent images. Additionally, repeated exposure to unattenuated X-ray beams can temporarily reduce the sensitivity of the image receptor in that region.

*Performance criteria*

Image retention of less than 0.5% between exposures.

#### Distance accuracy

*Frequency: Acceptance and software reload/upgrade*

*Rationale*

Software distance indicators (calipers) must be accurate as they are used to measure clinical pathology. In addition, images must be free from any distortion as this may result in misdiagnosis. Distortion can arise from incorrect DICOM data, software malfunction or hardware issues.

*Performance criteria*

Software displayed/measured distances should be within ± 2% and must be within ± 4% of actual distance.

#### Image receptor resolution (MTF)

*Frequency: Commissioning and 2-yearly thereafter*

*Rationale*

The resolving power of image receptors degrades as the frequency increases. A limit is imposed on the resolving power of any image receptor by the Nyquist frequency. To quantify the spectral characteristics of the resolving power of an image receptor, the IEC [17] proposes measuring the modulation transfer function (MTF) using a tungsten edge placed directly on the image receptor [17]. The beam conditions are according to RQA5 [18] with no scatter.

*Performance criteria*

50% MTF should not reduce by more than 0.4 cycles.mm^−1^ and must not change by more than 0.2 cycles.mm^−1^ compared to baseline measurement.

#### System resolution (sMTF)

*Frequency: Commissioning and 2-yearly thereafter*

*Rationale*

The system MTF contains the same information as the Image receptor MTF but includes more clinically realistic factors such as focal spot, geometric blurring and contrast reduction due to scatter.

*Performance criteria*

50% sMTF should not reduce by more than 0.4 cycles.mm^−1^ and must not change by more than 0.2 cycles.mm^−1^ compared to baseline measurement.

#### Image receptor noise power spectrum (NPS)

*Frequency: Commissioning and 2-yearly thereafter*

*Rationale*

The total noise property of an image receptor is an amalgamation of several sources of noise, with each contributing source displaying different spectral noise characteristics. Spectral noise characteristics can be quantified by measuring the noise power spectrum (NPS). The normalised noise power spectrum (NNPS) includes the number of incident quanta or effectively, the distribution of X-ray quanta.

*Performance criteria*

There should be no significant change in magnitude or shape of NPS spectra compared to baseline.

#### System noise power spectrum (sNPS)

*Frequency: Commissioning and 2-yearly thereafter*

*Rationale*

The system NPS contains the same information as the image receptor NPS but includes more clinically realistic factors such as grid scatter rejection.

*Performance criteria*

There should be no significant change in magnitude or shape of sNPS spectra compared to baseline.

### Peripherals

#### Kerma-area product (KAP) meter accuracy

*Frequency: Acceptance and 2-yearly thereafter*

*Rationale*

KAP meter accuracy is essential for patient dosimetry and for patient dose surveys.

*Performance criteria*

Displayed KAP value must be within 20% of measured KAP, and should be within 10%, for a clinical range of kV_p_ and collimations.

#### X-ray beam dimension scale

*Frequency: Acceptance and 2-yearly thereafter*

*Rationale*

Display of the X-ray beam dimensions should be accurate for all beam dimensions and FIDs.

*Performance criteria*

Indicated field size should be within 5% of measured value and must be within 10%.

#### Focus-to-image-receptor distance (FID) accuracy

*Frequency: Acceptance and as required thereafter*

*Rationale*

FID accuracy ensures that a correct distance is used for both dosimetry and image quality purposes.

*Performance criteria*

Displayed FID must be within 1% of measured. E.g. ± 1 cm at 1 m.

#### Collimation method

*Frequency: Acceptance*

*Rationale*

All new systems must have adjustable multi-leaf collimators, a light field indicating collimated area, and where programmable automatic collimation is provided, the operator must have the ability to manually override the automated selection.

*Performance criteria*

System must have adjustable multi-leaf collimators.

System must have a light field indicating the collimated area.

System must allow manual override of automated collimation.

#### Minimum focus-to-skin distance (FSD)

*Frequency: Acceptance*

*Rationale*

Exposures performed at an excessively short FSD can have high patient radiation dose and a high level of geometric blurring. Having a physically restricted minimum FSD ensures that exposures cannot be inadvertently taken with an inappropriately short FSD.

*Performance criteria*

The minimum FSD must be ≥ 200 mm.

#### Exposure switch location

*Frequency: Acceptance*

*Rationale*

The location of the exposure switch is a large practical determinant of radiation safety practices for operators.

*Performance criteria*

The exposure switch must be arranged so that the radiation apparatus can be operated from either:

- Behind a protective barrier; or
- A distance of at least 2 m from the X-ray tube

#### Initiation and termination of exposure

*Frequency: Acceptance*

*Rationale*

X-ray systems require a safety mechanism to identify when an X-ray exposure is underway, as there are no other sensory cues to alert people to the presence of radiation. There must also be means to terminate the exposure at any time.

*Performance criteria*

There must be conspicuous visible indication when the X-ray tube is energised.

There must be conspicuous audible indication either for:

- The duration of the X-ray exposure; or
- To identify the termination of the X-ray exposure

The exposure switch must be Deadman, and exposure must be able to be terminated at any time by the operator. Only one exposure can be initiated by a single actuation of the switch.

#### Indication of radiographic technique factors

*Frequency: Acceptance*

*Rationale*

X-ray operation is a combination of many complex interrelated factors. It is important that factors related to patient imaging are readily available for the operator to confirm prior to imaging, and are stored with the patient images.

*Performance criteria*

The X-ray system clearly displays the following exposure parameters when selected:

- The kV_p_
- The FID where an integrated bucky exposure is being performed
- The mAs and/or the mA and exposure time for manual exposures
- The AEC chambers selected for AEC exposures, if applicable
- The back-up mAs for AEC exposures
- The KAP, if available

Exposure factors relevant to patient dose calculations must be recorded in the DICOM header.

There must be a clear indicator to reflect system on/off status.

#### Indication of X-ray tube in operation

*Frequency: Acceptance*

*Rationale*

X-ray systems can have multiple X-ray tubes. The desired X-ray tube is carefully selected to ensure appropriate patient exposure and image quality based on patient and active X-ray tube positioning. Incorrect X-ray tube selection can lead to incorrect patient exposure, inadequate image quality, and/or unintentional exposure of others. It is essential for a user to be able to identify the active X-ray tube prior to making an exposure.

*Performance criteria*

For X-ray systems with more than one X-ray tube, there must be a conspicuous display indicating the active X-ray tube. Must be active during the entire duration of the exposure.

#### Indication of focal-spot location

*Frequency: Acceptance*

*Rationale*

The location of the X-ray tube focal spot is required for FID accuracy and minimum FSD checks.

*Performance criteria*

The location of the focal spot is clearly indicated in an easy to access location.

## Appendix 1: Summary of recommendations for facility QC procedures

**Table:** 

Test	Frequency	Tolerance	Key procedure elements	Record keeping	Recommended/optional
Detector calibration	As required	Manufacturer’s tolerance	To be performed according to the manufacturer’s specifications	Records to show: Calibration date Person performing the calibration Equipment identification Results and comments	Recommended
System constancy	Monthly	mAs ≤ ± 20% of Baseline or ≤ ± 0.2 mAs whichever is greater KAP ≤ ± 15% of Baseline EI ≤ ± 20% of Baseline MPV ≤ ± 20% of Baseline SD of MPV ≤ ± 20% of Baseline	1. Place the manufacturer’s calibration filter at the X-ray tube collimator 2. Set the FID to match the focal distance of the most commonly used grid 3. Set the collimation to just cover the image receptor 4. Perform an exposure under AEC control (all AEC chambers selected, 0 density) at a clinically relevant kV_p_ 5. Analyse a “for processing” image for each image receptor 6. Images can be extracted for processing, sent to PACS or analysed at the AWS	Control charts and records to show: Test date Person performing the test Equipment identification Plots of mAs, KAP, EI, and MPV Results and comments	Recommended
Image uniformity and artefact evaluation	Monthly	There must be NO: Significant shaded or bright areas Regions of altered noise appearance Gridlines or bucky structures Bright or dark pixels Structural damage to detector Radiopaque markers Residual signal from previous imaging Stitching or registration issues Significant encroachment of collimators into the field of view Image receptor electronics	1. Visually inspect a “for processing” image 2. Use a narrow window width and appropriate window level setting 3. Zoom and pan functions may be utilised	Records to show: Test date Person performing the evaluation Categories for evaluation Results and comments	Recommended
Modality display QC	Quarterly	Greyscale ramp bars are continuous All high contrast resolution patterns are visible No smearing at black/white transitions Squares of different shades are distinct 5% and 95% squares are clearly visible against 0% and 100% background No artefacts visible At least 11 letters are visible from “QUALITY CONTROL” i.e. “QUALITY CONT”	1. Display a TG18-QC test pattern 2. Ensure acceptable viewing conditions 3. Set the window-width to maximum and the window-level to half maximum 4. Evaluate checklist provided	Records to show: Test date Person performing the test Display identification Display settings Results and comments	Recommended
Mechanical and peripherals inspection	Quarterly	Cables are free of breaks, kinks, or knots Interlocks and brakes are functional Table, tube and bucky move smoothly Control panel switches, indicator lights and meters are functional System on/off status, image receptor loading, and operator-selected values are indicated on the control panel and are active during the entire duration of the exposure Light field is functional with adequate intensity Collimator is clean and free of dust Current technique charts are displayed near the control panel General area is clean, no oil leaks around the tube and generator X-ray tube and generator model and serial numbers are clearly marked and legible (if inaccessible, serial numbers must be displayed elsewhere e.g. in a file or label drawer) Operator has clear view of the patient from the control window Radiation warning signs displayed at all entrances to the room Warning lights are functional (if applicable) Cassette localisation/auto-collimation and locks functional Centring and FID detents are functional Distance displayed on the collimator is accurate System displays the selected AEC region(s) Exposures can be made at least 2 m from a mobile X-ray system or a protective barrier is fitted which covers the full height and width of the operator Exposure can be terminated at any time by the operator (dead-man switch)	Visually inspect the system for obvious signs of physical damage or wear and tear that could impact clinical use using the checklist provided	Records to show: Inspection date Person performing the inspection Results and comments	Recommended
X-ray/light field alignment	Quarterly	≤ ± 1% of the FID	To assess the accuracy and positioning of the alignment of the radiation and light beams: 1. Set an FID of 100 cm to the image receptor surface 2. Place four radio-opaque markers at the edges of the light field 3. Make an exposure using suitable low exposure factors 4. On the image, measure the distance from each edge of the radio-opaque markers to the edge of the radiation field	Records to show: Test date Person performing the test Equipment identification Results and comments	Recommended
Reject image analysis	Quarterly	Adult: between 5 and 10% Paediatric: between 3 and 7%	1. Extract data on rejected images 2. Categorise images if necessary 3. Calculate reject rate as number of rejected images divided by total number of images	Records to show: Test date Person performing the test Equipment identification Results and comments	Recommended
General X-ray quality meeting	Quarterly	Meetings held	Hold regular general X-ray review meetings to discuss facility QC, incidents, image quality complaints, repeat/reject analysis, radiation dose audits/trends etc	Meeting minutes	Recommended
Data validation	6-monthly and with software change	The following DICOM elements are recorded and displayed accurately: Facility ID/name Patient unique identifier Patient name Patient DOB Study date Study time Radiographic technique factors (kV_p_, mAs) Dose metrics (KAP, RAK) Exposure Index	View displayed details as well as DICOM header elements	Records to show: Test date Person performing the test Equipment identification Results and comments	Recommended
Dose and EI audit	Annually	Audit performed	Compare local dose and EI levels to published references and/or with appropriate equipment locally, looking at the machine, examination, view and operator, to identify the distribution of radiation dose and EI within a facility	Records to show: Audit date Person performing the audit Equipment identification Results and comments Trend analysis Recommendations for intervention if required	Recommended
Maintenance and fault logging	Ongoing	All maintenance and faults are recorded	Record all maintenance and fault activity	Records to show: Fault date/time Person logging the fault Equipment identification Description of the fault Actions taken Final result/follow-up	Recommended

## Appendix 2: Summary of recommendations for acceptance testing

**Table:** 

Test	Tolerance	Recommended/optional
X-ray tube and generator		
Tube output repeatability	The coefficient of variation of the X-ray output from a series of not less than 5 consecutive exposures must not exceed 0.05	Recommended
Tube output linearity	For any two mA or mAs settings │X1–X2│ ≤ 0.1 (X1 + X2) X1 = X-ray output per mAs (or mA for fixed exposure time) at setting 1 X2 = X-ray output per mAs (or mA for fixed exposure time) at setting 2	Recommended
Tube output	20–80 µGy/mAs at 80 kV and ≥ 2.5 mm Al Total Filtration, at 1 m	Optional
Tube voltage accuracy	The kV_p_ accuracy for kV_p_ settings across the clinical range must not exceed ± 5%	Recommended
Filtration	HVL ≥ 2.9 mm Al at 80 kV_p_ or meet IEC requirements [15] For systems installed prior to 2008, HVL > 2.3 mm Al at 80 kV_p_	Recommended
Timer accuracy	Measured time must be within ≤ 10% of indicated time for times ≥ 100 ms. Measured time must be within ± (10% + 1 ms) for times < 100 ms. Test should not be performed for times any lower than 20 ms as the measurement error of the equipment is too great	Recommended
Leakage radiation	Leakage < 1 mGy/hr at 1 m at maximum kV_p_ and continuous tube current	Recommended
Light/X-ray field alignment	< 1% of FID within light field < 1% of FID outside of light field	Recommended
Light/X-ray field congruence	Either: Centre of X-ray field and light field should coincide within 1% of FID Centre of X-ray field and light field must coincide within 1.5% of FID OR Centre of X-ray field and light field must coincide to within ± 3.8°	Recommended
X-ray to image receptor alignment	At maximum selectable FOV, X-ray field must extend to the edge of the active detector region and must not extend greater than 1% of FID beyond the edge of the image receptor	Recommended
Light field illuminance	Illuminance should be > 160 lx at 1 m Illuminance must be > 100 lx at 1 m	Recommended
Automatic exposure control		
AEC repeatability	The coefficient of variation of the X-ray output from a series of 5 consecutive exposures must not exceed 0.05	Recommended
AEC termination	Under AEC control, system must not allow exposures greater than 600 mAs	Recommended
AEC guard timer	If a guard timer is available, AEC controlled exposures must terminate before or at the set AEC guard timer	Recommended
AEC lateral chambers	The AEC system must control exposures such that the displayed mAs does not vary by more than 10% between individual AEC chambers for a consistent attenuation and set tube voltage	Recommended
AEC indication	System must display a visible indication of the selected image receptor and AEC chamber(s), and indicated selections must match the active chambers	Recommended
Image receptor		
STP	Relationship between image receptor incident air kerma and MPV must be verified as simple (e.g. linear, log or power) with R^2^ > 0.99	Recommended
Exposure Index (EI)	Using RQA5 beam quality. EI must be within 20% of IEC defined EI relationship: EI = 100 × image receptor incident air kerma (µGy)	Recommended
Artifact evaluation	No clinically significant artifacts on flat field image	Recommended
Uniformity	The maximum deviation between the STP-corrected central MPV and any other MPV should be less than 10%	Recommended
Defective DELs	No clinically significant visible defective DELs Number of defective DELs within manufacturers specification	Recommended
Distance accuracy	Displayed dimensions within 2% of actual dimensions	Recommended
Peripherals		
KAP meter accuracy	Displayed KAP value must be within 20% of measured KAP, and should be within 10%	Recommended
X-ray beam dimensions scale	Indicated field size should be within 5% of measured value and must be within 10%	Recommended
FID accuracy	Displayed FID must be within 1% of measured	Recommended
Collimation method	System has adjustable collimators Collimated FOV indicated by light field Automated collimator is able to be manually overridden to allow for smaller collimated field sizes	Recommended
Minimum FSD	Minimum FSD must not be less than 200 mm	Recommended
Exposure switch location	The exposure switch must be arranged so that the radiation apparatus can be operated from either: (a) Behind a protective barrier; or (b) A distance of at least 2 m from the X-ray tube	Recommended
Initiation and termination of exposure	Visual and audible indicator of exposure duration/termination Exposure switch is Deadman, and exposure can be terminated at any time by the operator. Only one exposure can be initiated by a single actuation of the switch	Recommended
Indication of radiographic technique factors	Relevant exposure parameters displayed on console and recorded in DICOM header, indicator for system on/off status	Recommended
Indication of X-ray tube in operation	Visible indicator of active X-ray tube (if more than one X-ray tube can be used)	Recommended
Indication of focal spot location	The location of the focal spot must be clearly and visibly indicated	Recommended
Mechanical inspection checklist	System shows no obvious signs of physical damage or significant wear and tear that could impact clinical use. Refer to 3.2.5 and checklist in Appendix 1	Recommended
Data validation	DICOM data populating accurately. Refer to 3.2.9 and checklist in Appendix 1	Recommended

## Appendix 3: Summary of recommendations for commissioning

**Table:** 

Test	Tolerance	Recommended/optional
AEC		
AEC sensitivity	Under clinical exposure conditions, the image receptor incident air kerma should be within ± 20% of the target air kerma, and be ≤ 3 µGy Investigations must be undertaken if the image receptor incident air kerma is > 5 µGy	Recommended
AEC reproducibility	Set baseline values for each exam	Recommended
kV_p_ variation	Exposure index normalised to image receptor incident air kerma must not vary by more than 20% across a range of clinically used tube voltages	Recommended
Image receptor		
Variance image	No significant variance defects are visible Retain image for future comparisons	Optional
Stitching	No clinically significant stitching	Recommended
Image retention	Image retention < 0.5%	Optional
Image receptor MTF	Set baseline	Recommended
sMTF	Set baseline	Optional
Image receptor NPS	Set baseline	Recommended
sNPS	Set baseline	Optional
User QC		
System constancy	KAP—set baseline mAs—set baseline EI—set Baseline MPV—set Baseline	Recommended
Modality display QC	Setup monitor QC procedure for facility QC	Recommended
Reject image analysis	Ensure that reject image analysis protocol has been established. Reject categories standardised. Data extraction and analysis method established	Recommended
General X-ray quality meeting	Ensure that General X-ray quality meeting terms of reference have been established with local facility	Recommended
Data validation	Ensure that access has been enabled for any data required for facility QC. This can include but is not limited to: Radiation Dose Structured Reports Reject analysis software Exposure logs	Recommended
Dose and EI audit	Ensure that dose and EI analysis protocol has been established and data extraction and analysis method established Ensure that appropriate target EI values have been applied to clinical protocols	Recommended
Maintenance and fault logging	Ensure that there is a standardised and documented method for logging faults and maintenance activity	Recommended

## Appendix 4: Summary of recommendations for routine (two-yearly) quality control

**Table:** 

Test	Tolerance	Recommended/optional
X-ray tube and generator		
Tube output repeatability	The coefficient of variation of the X-ray output from a series of not less than 5 consecutive exposures must not exceed 0.05	Recommended
Tube output linearity	For any two mA or mAs settings │X1 – X2│ ≤ 0.1 (X1 + X2) X1 = X-ray output per mAs (or mA for fixed exposure time) at setting 1 X2 = X-ray output per mAs (or mA for fixed exposure time) at setting 2	Recommended
Tube output	20–80 µGy/mAs at 80 kV and ≥ 2.5 mm Al Total Filtration, at 1 m	Optional
Tube voltage accuracy	The kV_p_ accuracy for kV_p_ settings across the clinical range must not exceed ± 5%	Recommended
Filtration	HVL ≥ 2.9 mm Al at 80 kV_p_ or meet IEC requirements [15] For systems installed prior to 2008, HVL > 2.3 mm Al at 80 kV_p_	Recommended
Timer accuracy	Measured time must be within ≤ 10% of indicated time for times ≥ 100 ms. Measured time must be within ± (10% + 1 ms) for times < 100 ms. Test should not be performed for times any lower than 20 ms as the measurement error of the equipment is too great	Recommended
Leakage radiation	Leakage < 1 mGy/hr at 1 m at maximum kV_p_ and continuous tube current	Recommended
Light/X-ray field alignment	< 1% of FID within light field < 1% of FID outside of light field	Recommended
Light/X-ray field congruence	Either: Centre of X-ray field and light field should coincide within 1% of FID Centre of X-ray field and light field must coincide within 1.5% of FID OR Centre of X-ray field and light field must coincide to within ± 3.8°	Recommended
X-ray to image receptor alignment	At maximum selectable FOV, X-ray field must not extend greater than 1% of FID beyond the edge of the image receptor	Recommended
Light field illuminance	Illuminance should be > 160 lx at 1 m Illuminance must be > 100 lx at 1 m	
AEC		
AEC sensitivity	Under clinical exposure conditions, the image receptor incident air kerma should be within ± 20% of the target air kerma, and be ≤ 3 µGy Investigations must be undertaken if the image receptor incident air kerma is > 5 µGy	Recommended
AEC repeatability	The coefficient of variation of the X-ray output from a series of 5 consecutive exposures must not exceed 0.05	Recommended
AEC reproducibility	Post-exposure mAs within ± 20% of baseline values for each exam	Recommended
kV_p_ variation	Exposure index normalised to image receptor incident air kerma must not vary by more than 20% across a range of clinically used tube voltages	Recommended
AEC termination	Under AEC control, system must not allow exposures greater than 600 mAs	Recommended
AEC guard timer	If a guard timer is available, AEC controlled exposures must terminate before or at the set AEC guard timer	Recommended
AEC lateral chambers	The AEC system must control exposures such that the displayed mAs does not vary by more than 10% between individual AEC chambers for a consistent attenuation and set tube voltage	Recommended
Image receptor		
STP	Relationship between image receptor incident air kerma and MPV must be verified as simple (e.g. linear, log or power) with R^2^ > 0.99	Recommended
Exposure index (EI)	Using RQA5 beam quality. EI is within 20% of IEC defined EI relationship: EI = 100 × image receptor incident air kerma (µGy)	Recommended
Artifact evaluation	No clinically significant artifacts on flat field image	Recommended
Uniformity	The maximum deviation between the STP-corrected central MPV and any other MPV should be less than 10%	Recommended
Variance image	No significant changes from baseline	Recommended
Defective DELs	No clinically significant visible defective DELs Number of defective DELs within manufacturers specification	Recommended
Stitching	No clinically significant stitching	Optional
Image receptor MTF	Baseline should be within ± 0.2 and must be within ± 0.4 cycles/mm for 50% MTF	Optional
sMTF	Baseline should be within ± 0.2 and must be within ± 0.4 cycles/mm for 50% MTF	Optional
Image receptor NPS	No significant changes in magnitude or shape compared to baseline	Optional
sNPS	No significant changes in magnitude or shape compared to baseline	Optional
Peripherals		
KAP meter accuracy	Displayed KAP value must be within 20% of measured, and should be within 10%	Recommended
X-ray beam dimensions scale	Indicated field size should be within 5% of measured value and must be within 10%	Recommended
User QC		
User QC	Performance checks have been performed at appropriate frequency Performance checks results are within limits, or actioned when outside of limits	Recommended
Dose and EI audit	Dose and EI are appropriate for facility. Where possible, compare to other systems within facility, and to similar systems with similar clinical workload from other facilities	Recommended
Maintenance and fault logging	Ensure that maintenance is occurring at an appropriate frequency Ensure that faults have been appropriately addressed	Recommended
General X-ray quality meeting	Ensure that actions required from the General X-ray quality meeting have been appropriately actioned	Recommended

## Appendix 5: Abbreviations and acronyms

**Table:** 

Abbreviation and acronym	Written form in-text
AAPM	American Association of Physicists in Medicine
ACPSEM	Australasian College of Physical Scientists and Engineers in Medicine
AEC	Automatic exposure control
AK	Air kerma
AK_ref_	Reference air kerma
ALARA	As low as reasonably achievable
AS/NZS	Australian Standards and New Zealand Standards
CoV	Coefficient of variation
CR	Computed radiography
DAP	Dose-area product (see also KAP)
DDI	Detector dose indicator (see also EI)
DI	Deviation index
DICOM	Digital Imaging and Communications in Medicine (standard)
DQE	Detective quantum efficiency
DR	Digital radiography
DRL	Diagnostic Reference Level
EI	Exposure index
FDD	Focus-to-dosimeter distance
FID	Focus-to-image-detector Distance
FOV	Field of view
FPD	Flat panel detector
FSD	Focal spot-to-skin distance
HVL	Half-value layer
ICRP	International Commission on Radiological Protection
IEC	International Electrotechnical Commission
IPEM	Institute of Physics and Engineering in Medicine
KAP	Airkerma-area product (see also DAP)
kV_p_	Kilovoltage peak
LC	Linearity coefficient
mA	Milliampere
mGy	Milligray
mSv	Millisievert
MTF	Modulation transfer function
NPS	Noise power spectrum
PEP	Patient equivalent phantom
PMMA	Polymethyl methacrylate
QA	Quality assurance
QC	Quality control
QMPS	Qualified Medical Physics Specialist
RANZCR	Royal Australian and New Zealand College of Radiologists
RDSR	Radiation dose structured report
ROI	Region of interest
SDD	Source-to-dosemeter distance
SNR	Signal-to-noise ratio
STP	Signal transfer property

## Data Availability

Not applicable.
